# Long-Term Prognosis after Surgery for Locally Recurrent Rectal Cancer: A Retrospective Study

**DOI:** 10.31557/APJCP.2021.22.5.1531

**Published:** 2021-05

**Authors:** Masato Nishimuta, Kiyoaki Hamada, Yorihisa Sumida, Masato Araki, Kouki Wakata, Tota Kugiyama, Ayako Shibuya, Shintaro Hashimoto, Keisuke Ozeki, Shigeyuki Morino, Soitiro Kiya, Masayuki Baba, Akihiro Nakamura

**Affiliations:** *Department of Surgery, Sasebo City General Hospital, 9-3 Hirase, Sasebo, Nagasaki, Japan. *

**Keywords:** Rectum cancer, local recurrence, long-term prognosis

## Abstract

**Objective:**

Resection is usually recommended for locally recurrent rectal cancer (LRRC) for which R0 resection is possible, but its suitability varies by individual patient risk. Here, we report outcomes of resected LRRC in our hospital.

**Methods:**

We retrospectively evaluated short- and long-term results of 33 patients who underwent resections for LRRC from January 2003 to December 2019.

**Results:**

At the initial surgeries for these 33 patients, their disease stages at that time were Stage I: n=2, Stage II: n=12, Stage III: n=11, Stage IV: n=6, and unknown: n=2. Patients with Stage IV disease at their initial surgeries underwent radical one-step or two-step procedures. Metastasis to other organs was observed in 5 patients at the their initial LRRC diagnoses. At the LRRC surgeries, 7 patients received palliative surgeries; 26 received intent-to-treat resections, of which 17 were R0 resections. All-grade postoperative complications were observed in 11 patients, including 1 surgery-related death. Five-year overall survival rates were all cases: 38.4%; R0 group: 52.3%, R1 or R2 group: 19.4%, and palliative surgery group: 0%. The R0 group thus had significantly better prognosis than other patients (P = 0.0012). Eleven patients in the R0 group (64.7%) suffered re-recurrences but some patients achieved long-term survival through chemotherapy, radiation therapy, and surgery for metastasis to other organs, even after re-recurrence.

**Conclusion:**

Long-term prognosis after surgery for LRRC was significantly better for patients with R0 margins. Multimodal treatments may greatly improve survival for patients who suffer re-recurrences after local recurrence resections.

## Introduction

Local recurrence of rectal cancer (LRRC) after radical surgery is relatively common (Kobayashi et al.,2007), and is usually treated with R0 resection (The curative resection was defined as R0 resection.) (Bhangu et al., 2012). However, the R0 resection rate varies widely (37% to 63%) due to anatomical complexity and differences in institutional indications for this surgery (Nielsen et al., 2012; Ghouti et al., 2015; Harris et al., 2016). Radical resection for LRRC must be carefully considered, as it is highly invasive, has a high postoperative complication rate, and has a large effect on the postoperative quality of patients’ lives (such as the need for artificial anus). Use of multimodal treatments besides surgery for LRRC is increasing.

At our hospital, we have been actively performing resections for LRRC except for sacral resections. We have analyzed patients’ short-term and long-term outcomes to better inform our treatment policy for LRRC.

## Materials and Methods

*Patients*

This study included 33 patients who underwent radical resection for LRRC at our institution from January 2003 to December 2019. For each of the 33 patients, we obtained the following data: age, sex, tumor factors of the initial surgery, period from initial surgery to recurrence, surgical procedure and data, postoperative complications, adjuvant treatment or additional treatment, re-recurrence, and long-term outcome.

After the initial surgery for rectal cancer, each patient was carefully followed up. We diagnosed local recurrence mainly by CT imaging. Local recurrences were classified into anterior, posterior, lateral or anastomosis recurrences, on the basis of their locations. Surgeries were planned when each local recurrence was diagnosed, with consideration for whether radical resection was possible and the patient’s general condition. Surgeries for LRRC were roughly divided into intent-to-treat resections and palliative surgery. In resection cases, curative resection was defined as R0 resection, resection with histological remnants was defined as R1 resection, and resection with macroscopic remnants was defined as R2 resection. Palliative surgeries were defined as surgery aimed at relieving intestinal obstruction. After surgery, patients were followed up, and received chemotherapy and/or radiation therapy, or palliative treatment, depending on their general condition and the residual condition of their tumors. The study design was approved by the Ethics Review Board of our institution. Each patient provided informed consent for data collection.

*Statistical analysis*

Continuous data are expressed as means ± standard deviations. Staging used UICC TNM classification (Brierley et al., 2017). The endpoint of this study was overall survival (OS) at 5 years after surgery, defined as the time between surgery to the date of death from any cause or the last follow-up, and analyzed using the Kaplan–Meier method. The log rank test was used to assess statistical significance. P < 0.05 was considered significant. Data analyses were all carried out using JMP13 for Windows software.

## Results

*Patient demographics and initial surgical data*

This cohort included 24 men and 9 women, whose mean age at the time of surgery for LRRC was 65.7±9.8 years. UICC calcification was used to for patients’ TNM staging (T2: n=4, T3: n=19, T4a: n=5, T4b: n=3, unknown: n=2; N0: n=17, N1: n=10, N2: n=4, N3: n=0, unknown: n=2). Pathological staging was I: n=2, II: n=12, III: n=11, IV: n=6, and unknown: n=2. For all Stage IV cases, R0 surgery was performed by resection of distant metastases in one- or two-step surgeries. Adjuvant chemotherapy was given in 14 cases and not given in 19 cases (Table 1).

*Surgery for local recurrence*

The mean period from initial surgery to recurrence was 548.2±360.1 days. Of the 33 patients who underwent surgery for LRRC, 26 underwent resections of their LRRC and 7 underwent palliative surgeries. Among the 26 patients who received resections, 17 had R0 resections, 2 had R1 resections, and 6 had R2 resections. We classified patients into the R0 group, the R1 or R2 group, and the palliative surgery group.

Details of recurrence locations, surgical procedures and postoperative complications are shown in Table 2. Postoperative complications (all Clavien–Dindo grades) were observed in 11 patients (surgical site infection: n=7, intestinal obstruction: n=1, pelvic abscess: n=1, anastomosis leakage: n=1, neurogenic bladder: n=1). Eight patients suffered grade ≥3a complications. One patient died of surgery-related causes.

We compared the R0 group with the non-R0 groups (the R1–R2 group, and palliative surgery group) by sex, age, factor of initial surgery, local recurrence location, and period until recurrence (Table 3). The R0 group and the non-R0 groups did not significantly differ in these factors, but the R0 group tended to have more Stage 3 patients, and the non-R0 group had more Stage 2 patients.

*Long-term outcome*

**Figure 1:**
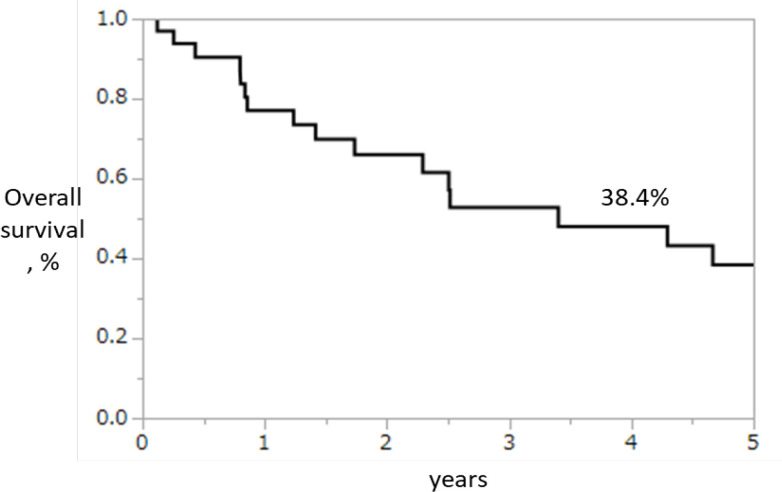
The 5-Year OS Rates of All 33 Patients

**Figure 2:**
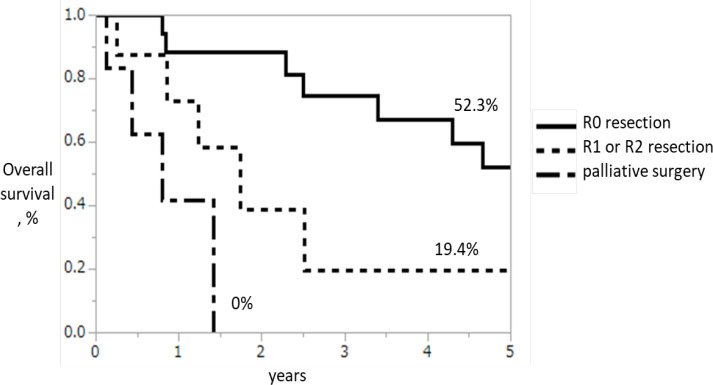
The 5-Year OS Rates Analyzed by Classifying into Groups R0 Group, R1–R2 Group, and Palliative Resection Group. The curative resection was defined as R0 resection, resection with histological remnants was defined as R1 resection, and resection with macroscopic remnants was defined as R2 resection

The 5-year OS rates were all 33 patients: 38.4% (Figure 1), R0 group: 52.3%, R1–R2 group: 19.4%, and 0% palliative resection group: 0%; the R0 group had significantly better 5-year OS than the other groups (P=0.0012; Figure 2). Re-recurrence after R0 resection was seen in 11 patients (64.7%), but some of these patients achieved long-term survival through chemotherapy, radiation therapy, and surgical treatment for metastasis to other organs, even after re-recurrence (Table 4).

**Table 1:** Patients Characteristics and Tumor Information of Initial Surgery (n=33). *Pathological data according to UICC classification

age (years)	67.5±9.8
Gender (male/female)	24/9
T factor (2/3/4a/4b/unknown)*	4/19/5/3/2
N factor (0/1/2/unknown)*	17/10/4/2
Stage (I/II/III/IV/unknown)*	2/12/11/6/2
adjvant chemotherapy (+/-)	14/19

**Table 2:** The Data of Surgery for Local Recurrence (n=33)

	R0 resection n=17	R1 or R2 resection n=9	palliative surgery n=7
Location of localy reccurence anterior/posterior/lateral/anastomosis	3/1/2/11	1/3/1/4	1/1/0/5
Surgical procedure	Low anterior resection 1	Hartmann's procedur 3	Colostomy constoruction 4
	Hartmann's procedure 3	Miles procedure 6	Small bowel resection 2
	Miles procedure 12		Intestinal bypass 1
	Total pelvic exenteration 1		
Postoperative complication all grade (≥grade 3a)	9 (6)	2 (2)	0 (0)
Detail of Grade3a postoperative complication	Surgical site infection 5	Pelvic abcess 1	-
	Neurogenic bladder 1	Anastomosis leakage 1	

*Grading was defined for Clavien-Dindo classification

**Table 3:** Comparison of Characteristic, Factor of Initial Surgery and Information of Locally Recurrences between R0 Group and Non-R0 Group

	R0 group n=17	non-R0 group n=16	p value
Gender (Male/Female)	12/5	12/4	0.78
age (Years)	65.2±9.0	69.9±10.4	0.18
Factor of initial surgery			
T factor (2/3/4a/4b)*	3/10/2/1	1/9/3/2	0.81
N factor (0/1/2)*	7/7/2002	10/3/2002	0.54
Stage(I/II/III/IV)*	1/5/9/1	1/7/2/5	0.051
Location of locally recurrences (anterior/posterior/lateral/anastomosis)	1/3/2/11	4/2/1/9	0.37
Period until recurrence (days)	580.6±343.5	504.9±388.1	0.57

*, Pathological data according to UICC classification. Unknown data were excluded

**Table 4:** Detail of Re-Recurrence Cases after R0 Resection for Local Recurrence

case	age	gender	period until local recurrence (days *1)	surgery for local recurrence	adjuvant therapy	location of re-recurrence	period until re-recurrence (days *2)	therapy for re-recurrence	outcome (days *3)
1	66	F	408	LAR		local	189	CT	death (1076)
2	50	M	146	Miles		local	104	RT	death (293)
3	56	M	779	Miles		local	441	RT	death (916)
4	73	M	726	Miles		lymph node	359	BSC	death (1243)
5	75	M	1160	Miles		liver	225	surgery	death (1569)
6	76	M	741	Miles		local	706	BSC	death (839)
7	70	M	146	Miles	FOLFOX	lung	1086	CT	death (1706)
8	66	F	386	Miles	capeOX	local	249	CRT	death (1922)
9	71	M	642	Hartmann	S-1	local and lung	1814	CT	death (2377)
10	76	M	639	Miles		lymph node	71	CT	death (306)
11	54	M	995	TPE		lung	316	surgery	alive (895)

LAR, low anterior resection; TPE, total pelvic exenteration; CT, chemotherapy; RT, radiation therapy; CRT, chemoradiation therapy; BSC, best supportive care; *1, From initial surgery; *2, From surgery for local recurrence;*3, Survival days from surgery for local recurrence

## Discussion

LRRC is common in patients with rectal cancer (Kobayashi et al., 2007). In this study, we investigated surgical treatment for LRRC, which has been shown to be the most prudent current treatment for most LRRC cases (Bhangu et al., 2012; Uehara et al., 2015; Harris et al., 2016). However, resection for LRRC is often extremely difficult: adhesions may remain from the initial surgery, scarring may make peeling and setting of the excision line more challenging, and the pelvic anatomy is complex. Both this current report and a previous study show poor prognosis after R2 surgery, which indicates that resection should be avoided if R0 margins are not expected (Pellino et al., 2015). Therefore, an optimal surgical plan for LRRC requires accurate judgment as to whether resection is possible based on imaging and understanding of pelvic anatomy. We mainly used CT to diagnose local recurrence. Some studies have also found that magnetic resonance imaging and positron emission tomography are useful for diagnosing local recurrence (Schaefer and Langer., 2001; Zhang et al., 2009; Chew et al., 2013), and we auxiliary used for diagnosis. Because we did not perform combined sacral resections, we judged that radical resection was possible in the absence of sacral invasion and distant metastasis. If radical resection was preoperatively or intraoperatively judged to be infeasible, palliative surgery was performed, followed by postoperative chemotherapy, radiation therapy or best supportive care.

In our study, R0 resection was associated with good prognosis. Previous studies have shown that R0 resection is necessary to improve the prognosis of LRRC (Saito et al., 2003; Kanemitsu et al., 2010; Bhangu et al., 2012; Uehara et al., 2015; Harris et al., 2016). In our analysis, the survival rate for R0 resection was equivalent to those in previous studies. In recent years, multimodal treatment has also been shown to be increasingly useful; in particular, some reports have shown preoperative radiotherapy to be effective (Chew et al., 2013; Ogawa et al., 2015; Vermaas et al., 2015; Holman et al., 2017). Another report found that heavy ion radiotherapy led to prognosis equivalent to surgery, and it may be an important treatment option going forward (Yamada et al., 2016). In our study, 11 patients had re-recurrences after R0 resections, including 7 with local re-recurrences, 4 with lung metastases and 2 with lymph node metastases (with duplication). No consensus on postoperative adjuvant therapy to prevent re-recurrence after local recurrence resection is available. However, three patients achieved relatively long-term survival from adjuvant chemotherapy after local recurrence resection. Some patients also achieved long-term survival by excising lung metastases and receiving chemotherapy or radiation therapy, even after re-recurrence. We consider multimodal treatment (depending on the patient’s condition) important for better prognosis.

This study has some limitations. First, it has a small cohort, all treated at a single institution. Since LRRC often has different policies depending on each institution, we considered that a single institution trial is desirable even if the sample number is small. Second, we did not include patients who were treated only with chemotherapy and/or radiation. Comparisons between surgical and non-surgical treatments may be needed to find the true usefulness of surgical treatment, particularly in light of the great advances in chemotherapy and radiotherapy in recent years.

In conclusion, among patients with LRRC, the long-term prognosis of those who had R0 surgical margins was significantly better than those who did not. Also, our results indicate that multimodal treatments improve chances for long-term survival for patients who suffer re-recurrences after LRRC resections.

## Author Contribution Statement

Masato Nishimuta and Kiyoaki Hamada contributed to the planning and composition of this clinical study. Masato Araki, Kouki Wakata, Kiyoaki Hamada, Tota Kugiyama, Ayako Shibuya, Shintaro Hashimoto, Keisuke Ozeki, Shigeyuki Morino, Soitiro Kiya, Masayuki Baba and Akihiro Nakamura gave critical opinions on data analysis and manuscript preparation. All authors approved the final article. 
